# The Fate of Intranasally Instilled Silver Nanoarchitectures

**DOI:** 10.1021/acs.nanolett.2c01180

**Published:** 2022-06-30

**Authors:** Agata Zamborlin, Maria Laura Ermini, Maria Summa, Giulia Giannone, Valentina Frusca, Ana Katrina Mapanao, Doriana Debellis, Rosalia Bertorelli, Valerio Voliani

**Affiliations:** † Center for Nanotechnology Innovation@ NEST, Istituto Italiano di Tecnologia, Piazza San Silvestro, 12−56127, Pisa, Italy; ‡ NEST-Scuola Normale Superiore, Piazza San Silvestro, 12−56127, Pisa, Italy; § Translational Pharmacology, Istituto Italiano di Tecnologia, Via Morego, 30−16163, Genoa, Italy; ∥ Electron Microscopy Facility, Istituto Italiano di Tecnologia, Via Morego, 30−16163, Genoa, Italy

**Keywords:** silver, gold, antimicrobial resistance, lung infection management, biodistribution, communicable diseases, SARS-CoV-2, argyria

## Abstract

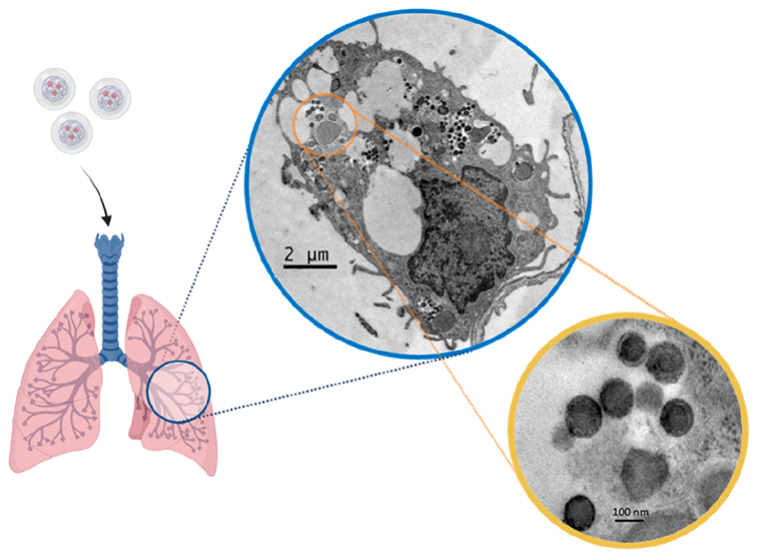

The intranasal administration of drugs allows an effective and noninvasive therapeutic action on the respiratory tract. In an era of rapidly increasing antimicrobial resistance, new approaches to the treatment of communicable diseases, especially lung infections, are urgently needed. Metal nanoparticles are recognized as a potential last-line defense, but limited data on the biosafety and nano/biointeractions preclude their use. Here, we quantitatively and qualitatively assess the fate and the potential risks associated with the exposure to a silver nanomaterial model (i.e., silver ultrasmall-in-nano architectures, AgNAs) after a single dose instillation. Our results highlight that the biodistribution profile and the nano/biointeractions are critically influenced by both the design of the nanomaterial and the chemical nature of the metal. Overall, our data suggest that the instillation of rationally engineered nanomaterials might be exploited to develop future treatments for (non)communicable diseases of the respiratory tract.

In the final advice 1618/20 of the Scientific Committee on Consumer Safety (SCCS) of the European Union, inhalation exposure to nanoparticles is considered “of the highest risk [with respect to other exposure pathways] because particulate materials generally tend to induce more harm to the respiratory system”.^1^ In the same advice and with a pre-emptive approach, the SCCS raises concerns over consumers safety from the use of products containing nanomaterials.^1^ At present, no final conclusion on the safety of nanomaterials has been drawn from the SCCS nor from the Scientific Committee on Emerging and Newly Identified Health Risks (SCENIHR) because of the lack of adequate biodistribution and safety data.^2^ In this scenario, it is of critical relevance to elucidate the fate of nano-objects once they are inhaled.

Among metal nanoparticles, silver nanoparticles (AgNPs) are the most employed for biomedical and commercial purposes owing to their antimicrobial properties.^3^ In clinical research, silver nanoparticles represent one of the most promising materials to overcome microbial drug resistance.^4^ Indeed, AgNPs exhibit multiple mechanisms of action, associated with their dissolution resulting from silver oxidation to Ag^+^_,_ that affect microbial walls, organelles, and biochemical pathways.^4^ Silver nanoparticles have only recently been recognized as the last-line of defense to reduce both bacterial burden and inflammatory response, even if already employed more than 100 years ago to treat septicaemia.^5^ Some silver nanoparticles effectively inhibit multidrug resistant strains with low tendency to generate further drug resistance.^6^ Moreover, AgNPs can bind spike proteins on viral capsids, blocking their interactions with human cell receptor.^7^ This mechanism may also help to eradicate SARS-CoV-2 pulmonary infection in its early phase, especially if nanoparticles are administered intranasally.^7^

The administration of pharmaceuticals via inhalation allows for a more efficient therapeutic action in the respiratory tract when compared to intravenous injection.^8^ Consequently, a lower amount of the therapeutic can be employed, hence reducing the risk of possible toxicity.^8^ Inhalation may improve the patient’s compliance since it offers a noninvasive delivery method that can be self-administered, resulting in a positive impact on the treatment efficacy.^9^

AgNPs can be found in commercial respirators, household water filters, catheters, cardiovascular implant, food packages, cosmetics, and textiles.^10^ Inhalation is deemed the main exposure route in workplaces where AgNPs are produced.^3,11^ In 2016, Weldon et al. suggested an occupational exposure limit (OEL) to AgNPs of 0.19 μg/m^3^ on the basis of investigations on subchronic inhalation in rats mimicking the occupational exposure.^12^ The OEL proposed by Weldon was established on 90 days of inhalation toxicity studies of Sung et al., and it was further confirmed by Hadrup et al. four years later.^13−15^ A “no observed adverse effect level” (NOAEL) of 100 μg/m^3^ was previously reported, according to histopathological effects and silver redistribution to the organs.^13^ However, by considering tissue dosimetry of the reviewed studies, Weldon et al. noted that liver was more sensitive to AgNPs than lungs, which supports the lower threshold proposed in their work.^12^

Nevertheless, a detailed assessment of the nano/biointeractions and of the metabolism of inhaled AgNPs has still, to date, to be completed. The large adoption of AgNPs raises concerns regarding the potential associated risks to their direct and indirect exposure after inhalation.^16,17^ When AgNPs are in contact with organic fluids, AgNPs release silver ions or bind to biomolecules. AgNPs’ ions release is called “Trojan Horse effect”.^10,18^ For example, Wen et al. found the intranasal instillation of silver ions to be more toxic than AgNPs administration at the same silver concentration and with the same exposure schedule.^19^ Nonetheless, it is still unclear whether the main responsible of the observed toxicity is nanoparticulate silver, released silver ions, or both.^10,11^ Indeed, theoretical studies suggest that AgNPs can penetrate in the respiratory tract and interact with lung macrophages, epithelial cells, and pulmonary surfactants, causing inflammation and even granulomatous lesions.^3,20^ Oxidative stress, DNA damage, and inflammation can be observed in the site of primary exposure, i.e. lungs, and in secondary organs as well.^10^ Effects on both primary and secondary organs can be related to particle size, route, and duration of exposure, dose, and end points of the experiments. Remarkably, while silver amount in all organs reduces over time, it shows a different trend in brain and testes.^10,11,21^ This tendency was observed both after AgNPs intranasal exposure by Wen et al., and oral administration by van der Zande et al. and Lee et al.^19,21,22^ Even if based on oral administration of AgNPs, these works provide interesting information that are complementary to those of intranasal and inhalation exposure studies, considering the inevitable ingestion of part of the intranasally administered dose. Indeed, AgNPs deposited in the upper respiratory tract are likely cleared by the mucociliary system into the gastrointestinal tract (GIT).^12^ From the data reported by SCENIHR, Weldon et al. suggested that <0.1–4% of the inhaled dose is absorbed by GIT.^12,23^ In this context, biokinetic assessments allow for the evaluation of the nanomaterial biosafety profile as well as the environmental exposure risk, finally unlocking the potential of AgNPs in oncology, communicable disease management, and other nonclinical fields.^16,24^

In order to clarify the fate of nanomaterials after intranasal instillation, we evaluate the *in vivo* absorption–distribution–metabolism–elimination–toxicity (ADMET) of silver nanoarchitectures (AgNAs). AgNAs are ultrasmall silver nanoparticles (<2 nm) embedded in biodegradable silica nanocapsules.^25^ Because of this rational design, the silica shell protects the inner functional core until it reaches the target, allowing the release of the metal directly in the place of action.^26^ Furthermore, thanks to their unique design and versatility, the nanoarchitectures are a significant model to evaluate and compare the bio/nanointeractions of noble metals.^27^ For assessing the toxicity and the biokinetics of AgNAs, we quantitatively evaluate the metal amount in the organs of treated mice, and we analyze lung and brain tissues by histological and electron microscopy investigations. AgNAs display a good excretion profile and an almost negligible accumulation in the main organs. By contextualizing our findings in light of the existing literature on AgNPs, intravenously injected AgNAs and intranasally instilled gold nanoarchitectures (AuNAs),^27−29^ we present here a critical evaluation regarding the interactions of hybrid metal nanoparticles with living organisms and add relevant information about the fate of inhaled ultrasmall-in-nano metal nanoparticles. We ultimately observe that the nanomaterial design and the dissolution of silver critically affect its biodistribution and clearance profile.

## Results and Discussion

Silver nanoarchitectures (AgNAs) are intrinsically sterile and biodegradable nanoplatforms composed of 150 nm hollow silica nanospheres embedding ultrasmall silver nanoparticles in a functional polymeric matrix.^30^ The silica shell allows (i) the protection of the inner materials, (ii) easy modification of the surface properties, and (iii) enhancement of ultrasound signals.^31,32^ The inner materials can be modulated to better fit the final application by changing the metal (gold, silver, platinum, or copper) or including active molecules, among which are drugs or dyes.^33,34^ AgNAs have actually demonstrated interesting features as catalysts and antibacterial agents.^25,30^ It is worth noting that the NAs’ family has been developed to avoid the issue of plasmon nanomaterial persistence after the action with a special focus in oncology.^26,35^

**Figure 1:**
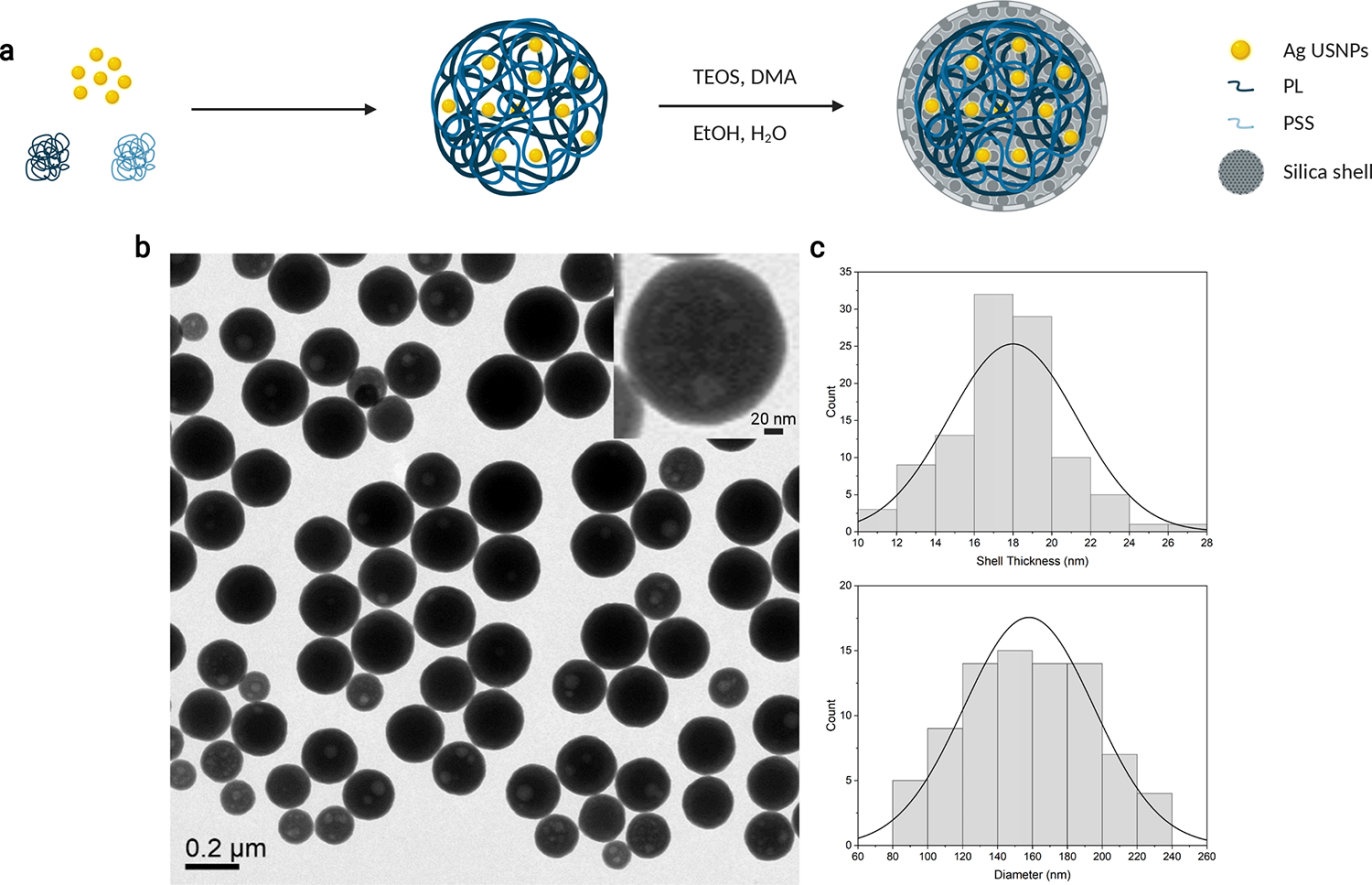
(a) Scheme of the synthesis of AgNAs. Silver seeds aggregate in a controlled fashion because of PSS and PL presence. The aggregates are the template for the silica shell formation. (b) Wide-area TEM image of AgNAs. Scale bar: 0.2 μm. The inset is a zoom on a single nanoarchitecture (Scale bar: 20 nm). (c) Size distribution of silica shell thickness (upper) and AgNAs diameter (bottom) made on at least 100 nanoparticles visualized with TEM. Nanoparticle diameter and shell size were analyzed using ImageJ.

AgNAs were synthesized following an optimized protocol.^27^ The final AgNAs size was 158.9 ± 36.6 nm with a silica shell thickness of 18 ± 3 nm (Figure 1). The metal loading (3%) was assessed by inductively coupled plasma-mass spectrometry (ICP-MS).

**Figure 2:**
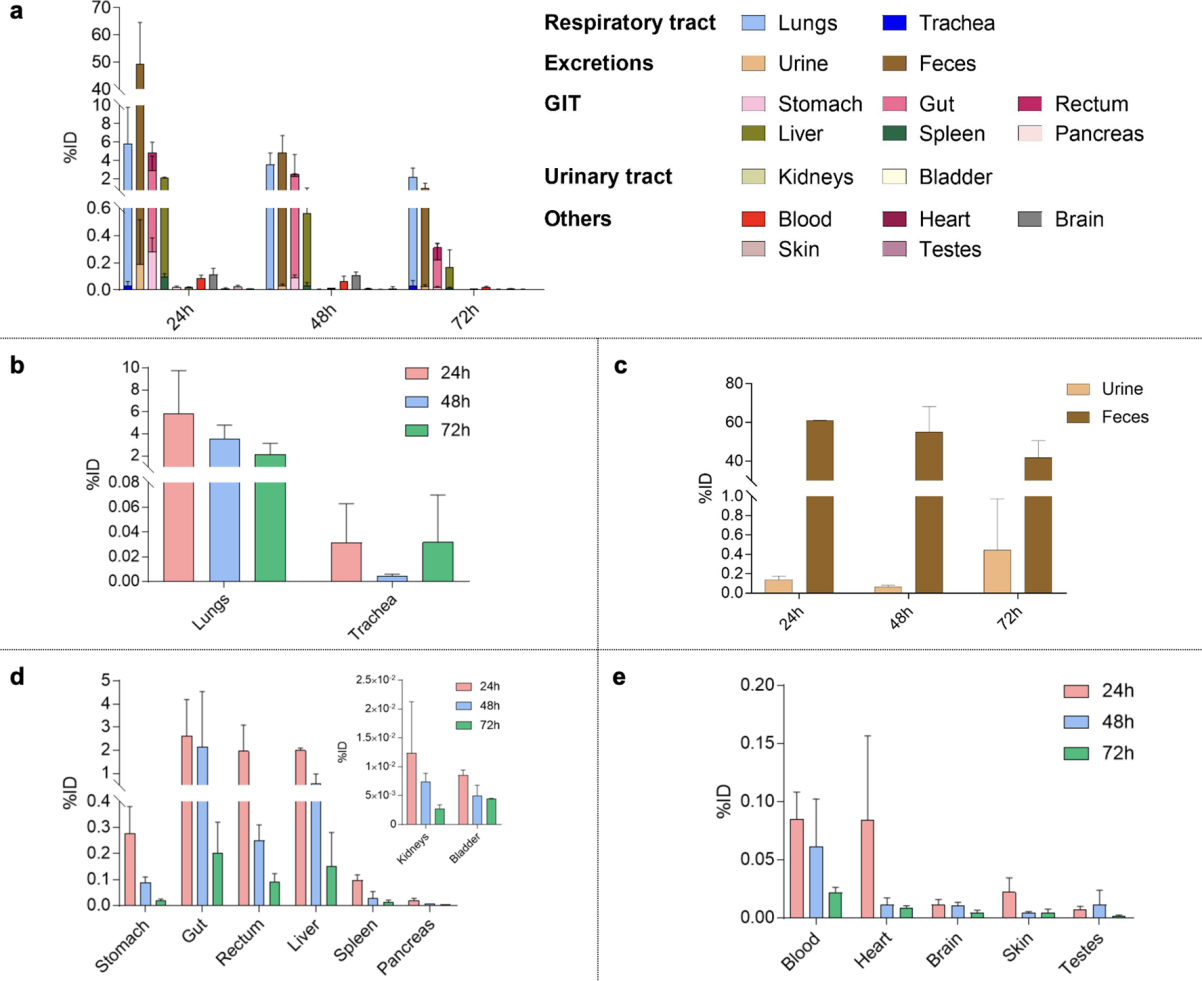
(a) Overview of silver biodistribution (%ID) in the excretions and in the main organs grouped by systems . b) Silver biodistribution (%ID) in the respiratory tract over 72 h . (c) Silver cumulative excretions (%ID) in feces and urine at different time points . (d) Silver biodistribution (%ID) in the GIT organs and hepatobiliary system. Inset: silver biodistribution (%ID) in the urinary tract . (e) Silver biodistribution (%ID) in blood and highly vascularized organs . Results are reported as mean ± standard deviation of n = 3 biological samples for each time point.

The biokinetics and excretion profile of AgNAs after intravenous (IV) tail-vein administration has been previously reported by our group using healthy CD1-*Foxn1*^*nu*^ mice, a model usually employed for heterotopic or orthotopic tumor xenograft.^27^ The same mouse model has consistently been employed to investigate AgNAs biokinetics after intranasal (IN) administration. The IN instillation is the most feasible pulmonary administration route in rodents.^28^ Indeed, it is well tolerated, may not require anesthesia, and can be easily translated to the clinical setting.^36^ Each mouse (roughly 30 g) was treated with 3 mg NAs/kg mouse, which was approximately 3 μg of silver. Previous studies suggest that this amount is within the acceptable dose range for particle inhalation in rodents.^37^ Urine and feces were collected every 24 h and the mice were sacrificed at 24, 48, and 72 h to collect the organs and quantify by ICP-MS the metal content (Figure 2).

The time points have been selected by considering both the NAs biodegradation kinetics and the potential mouse distress associated with single housing in metabolic cage.^28^ During the experimental period, no behavioral nor pathological changes on animals were observed upon daily inspection.

Twenty-four hours after the IN instillation, most of the administered metal was recovered in feces, in the gastrointestinal tract (GIT), and in the lungs (Figure 2a): 49% of inhaled dose (%ID), 4.8%ID, and 6%ID, respectively. Interestingly, only 0.03%ID of silver was recovered in the trachea (Figure 2b), confirming that AgNAs were not withheld. Mice are obliged nose breathers, and nasal deposition can be a concern.^37^ Indeed, particle size can be associated with significant nasal accumulation.^37^ Hence, our results suggest that AgNAs hydrodynamic diameter is suitable to avoid accumulation in the upper regions of the respiratory tract, confirming the importance of the design of AgNAs. In addition, AgNAs design avoids silver USNPs exhalation after administration.^38^

Silver content in lungs decreased by 50% and 70% over 48 and 72 h, corroborating the nonpersistence of the metal in the respiratory system. This metal reduction was not observed when AgNAs were administered intravenously as silver reached the lungs in very low amount (0.06% of the injected dose).^27^ Even when compared to intranasal gold nanoarchitectures (AuNAs) administration,^28^ inhaled silver NAs show less persistence. The inertness of gold avoids its dissolution and reduces the clearance efficiency in lungs. On the other hand, AgNAs release silver ions which are instrumental in accelerating lung clearance, thus reducing potential toxicity. Because of the peculiar design, AgNAs can promote transitory accumulation in the lungs while allowing for silver redistribution once the shell is eroded and improving the clearance profile of Ag USNPs (Figure S1). The reduction of silver content observed in the pulmonary tract and the importance of Ag dissolution agrees with several recent *in vivo* studies on inhaled AgNPs.^15^ Interestingly, Davidson et al. found a correlation between the size of inhaled AgNPs (20 and 110 nm) and their dissolution.^39^ In this regard, the clearance profiles of AgNPs reported by Hadrup et al. differ from our findings.^15^ This confirms that the elimination of silver from lungs is heavily affected by the design and the size of the silver nanomaterial. Indeed, Ag USNPs, which are inside AgNAs, are in the ultrasmall range. With a larger surface-to-volume ratio with respect to bigger AgNPs, Ag USNPs dissolve faster, by reducing the persistence time in the lungs. This behavior supports silver elimination and reduces the overall potential toxicity and the side-effects on lungs metabolic profile.^40^

One day after IN administration, the amount of silver in the urine (i.e., 0.19%ID) was much lower than in the feces (i.e., 49%ID) (Figure 2c). The preferential elimination of silver through feces and urines after inhalation or intratracheal instillation is also shown by Rosário et al. and Andriamasinoro et al.^40,41^ On the other hand, it is interesting to note that, compared to IV administration of AgNAs,^27^ the fecal excretion is still the main elimination pathway. Nevertheless, due to the collateral AgNAs ingestion during IN application and to the action of the mucociliary system^42−45^ the amount of metal detected in the feces after 24 h was higher than the one found at 24 h after IV administration. Ingestion likely leads to an excretion burst at the first time point because a significant amount of silver (Figure 2d) reaches the GIT (0.3, 2.6, and 1.9%ID for, respectively, stomach, gut, and rectum). The very low amount of silver in urine and the almost undetectable silver in the urinary tract (0.01%ID in the kidneys and 0.009%ID in the bladder) confirm the fecal excretion route and highlight the plausible low renal toxicity of AgNAs (Figure 2d inset).

The %ID in the liver (2%) and in the spleen (0.1%) 24 h after IN and the subsequent reduction trend corroborated the main GIT involvement on fecal excretion and the secondary role of hepatobiliary system (Figure 2d). Hepatic damage after pulmonary exposure is influenced by the dose, the administration method, the exposure schedule, and the nanoparticle size.^11^ The increase of exposure time can affect the liver as described by Sung et al. (13 weeks of inhalation exposure to 18 nm AgNPs).^13^ Oxidative stress and acute toxicity can be also observed after a single exposure to inhaled 10 nm AgNPs.^3^ Deterioration of the hepatic functionalities can be also associated with prolonged oral administration of AgNPs.^46^ In general, previous works indicate that the smaller the nanoparticles the less liver damage.^22,47^ Taken together, these data support that the design of AgNAs is responsible for the reduced risk of hepatic damages in our experiments. Indeed, the USNP size may reduce the accumulation and the redistribution to the liver. Silver showed a more pronounced persistence in the lungs compared to liver.^10^ This behavior may be related to the different morphology among the endothelium. Lungs are characterized by a continuous endothelium, whereas the blood vessels in the liver are rich in *fenestrae* (100–200 nm size).^48,49^ Since AgNAs diameter is around 150 nm, silver may enter the blood circulation from lungs mostly after the degradation of the silica shells (24–48 h).^27^ On the contrary, the crossing process in liver can occur even if the shell is not completely eroded due to the *fenestrae*. A similar behavior was observed with IN administration of AuNAs: gold clearance from the lung was slower compared to liver.^28^ Moreover, since Au does not dissolve, the reduction in the metal content is likely due to the direct escape of the USNPs from the lungs.^28^

The almost negligible silver amount in the blood and heart after 24 h (0.09%ID and 0.08%ID, respectively) suggests a low cardiotoxicity of the nanoarchitectures (Figure 2e). Moreover, after 72 h the silver content in the blood and heart decreased by 75% and 90%, respectively. It is worth to notice that also the intravenous injection of AgNAs did not induce significant silver content neither in the blood (0.13%ID at 48 h, 0.02%ID after 21 days) nor in the heart (0.02%ID at 48 h, 0.01%ID after 21 days).^27^ Other works in literature outline dose-dependent cardiovascular effects and exacerbation of cardiac ischemic-reperfusion associated with intratracheal instillation of 10–20 nm AgNPs.^50,51^ The different size and the ultrasmall-in-nano design of AgNAs can explain the different accumulation and persistence in the organs, hence the different toxicity.

Unexpectedly, the brain was a site of negligible silver accumulation after AgNAs IN administration: 0.01%ID after 24 h, and 0.004%ID after 72h (Figure 2e). Differently, the gold content in the brain after IN exposure to AuNAs was 2.5%ID at 24 h (despite the similarities of the external structure between AgNAs and AuNAs).^28^ Thus, AgNAs are not able to transiently accumulate in the brain through the olfactory nerve by passing the cribriform plate after IN administration as AuNAs.^28^ This finding is of particular interest as other studies report neurotoxicity mediated by reactive oxygen species associated with silver accumulation in the brain.^52,53^

Skin samples were collected at the different time points to investigate the potential silver accumulation in such an organ (Figure 2e). Indeed, chronic silver intake (approximately 2–4 g/day of silver) can lead to a pathological condition called argyria, which is characterized by a skin decolouration after metallic silver accumulation.^54^ This condition is associated with the affinity between Ag^+^ and cysteine-rich proteins, such as keratin and the N-terminal region of type VII collagen, and by the precipitation of Ag_2_S after UV light exposure.^55,56^ In our setup, no changes in skin color were observed and no signs of argyria were found. The silver collected in the skin was 0.02%ID/cm^2^ after 24 h, and it further decreased at 48 and 72 h.

Remarkably, silver biodistribution may be gender related.^57^ This is relevant when considering gender-specific toxicity, treatment performances, and effects on the reproductive system.^58^ The content of silver found in the testes 24 h from IN administration was 0.007%ID with a decreasing trend over time (Figure 2e). Hence, the possibility of reproductive toxicity after AgNAs IN exposure may be negligible. Other groups reported a significant accumulation of AgNPs in testes after intraperitoneal administration with subsequent influence on sperm quality.^59^ This highlights the importance of the administration pathway as well as the design of the nanomaterial on their biodistribution and potential toxicity.

The comparison between the current findings and our previous works on AuNAs biokinetics evaluation after IN administration allows us to understand the impact of chemical nature on metal biodistribution after IN exposure.^28^ First, the main excretory pathway is fecal with both nanoarchitectures, highlighting the importance of the administration route in the elimination profile. Indeed, AuNAs are mainly excreted through urines when injected intravenously.^29^ Furthermore, the chemical nature of the NAs core influences the excretion efficacy. Since gold oxidation does not occur, it is almost completely excreted (80%ID after 10 days). The cumulative silver collected in the organs and in the excretions 72 h after IN was instead 2.7%ID and 42%ID, respectively. This is associated with silver susceptibility to oxidation and conjugation to thiol-bearing proteins after NAs degradation. Silver ions could redistribute in the body in uncollected tissues among which are connective and adipose tissues. The oxidation can take place in the gastric and intestinal fluids, as well as in the mucoid secretions.^56,60^ The ionic species and the formed soluble complexes can exploit Na^+^ and Cu^+^ transporters to reach the blood. In this form, silver can bind thiol-bearing proteins, glutathione, and albumin with high affinity, enabling its redistribution to the body. It is worth noticing that the complex with glutathione also allows silver transport from hepatocytes to bile, which may also contribute to silver excretion.^53,61^

**Figure 3:**
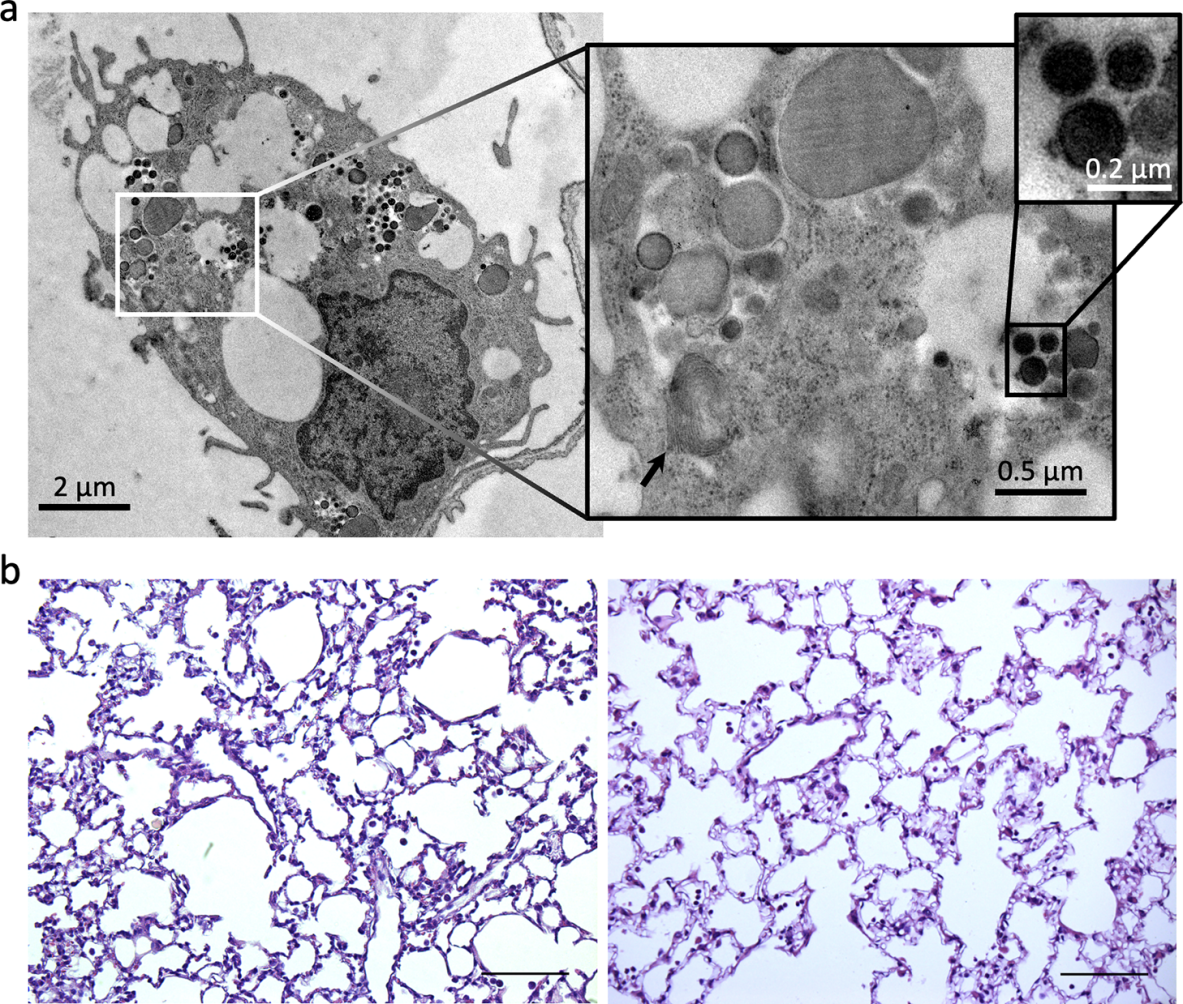
(a) TEM micrographs showing the presence of AgNAs in lungs 24 h after administration. Scale bars: 2, 0.5, and 0.2 μm. The black arrow highlights the presence of a lamellar body. (b) Histological analysis of lung tissues of control (left), and IN AgNAs (right) treated mice. Scale bar 100 μm.

The presence of AgNAs in lungs was directly observed with electron microscopy, confirming both AgNAs distribution in this organ and their degradation to the building blocks (Figure 3a and S1). We found that AgNAs were mainly localized in type II pneumocytes, that together with the type I are the cells that line the alveoli and comprise most of the inner surface of the lungs. No evident signs of damage were observed by TEM examination, particularly in the subcellular compartments: lamellar bodies, mitochondria and large vesicular nuclei were regular both in dimension and distribution. From the histological analysis of the lungs, no relevant damage was noticed at any time-point (Figure 3b). The lack of damages in the alveolar structure is associated with the intrinsic low toxicity of AgNAs as well as to the clearance of the building blocks. The lack of lung inflammation may appear in contrast with some previous findings reported in the literature.^3,62^ For example, Smulders et al. observed a significant uptake of 25 nm AgNPs by alveolar macrophages, followed by silver recomplexation with thiol-bearing molecules (metallothioneins).^63^ On the other hand, Braakhuis et al. observed that pulmonary inflammation is a consequence associated with both size-related lung deposition and dissolution rate of AgNPs (size, 15–410 nm).^62^ Thus, both the size and the chemical nature of the nanoparticles affect the interactions with the macrophages, leading to a different fate and toxicity. AgNAs are 150 nm silica nanocapsules when administered, limiting the alveolar deposition and the direct contact of the tissues and alveolar macrophages with the metal. Moreover, Ag USNPs reduce the persistence time in the lungs due to the high surface-to-volume ratio. These behaviors support silver elimination and reduce the overall potential toxicity. Besides histopathology of the lungs, bronchoalveolar lavage, oxidative stress markers, and cell proliferation should be further evaluated to better assess the pulmonary end points together with long-term analysis.^64^ On the other hand, our findings are further corroborated by TEM and histological analysis on AuNAs after IN (Figure S2) in which no signs of inflammation were recorded. These data further confirm that the design of nanomaterials strongly affects the distribution of the metals to lungs as well as their toxicity.

## Conclusions

The fate and the effects of a silver nanomaterial model, i.e. AgNAs, after a single dose instillation have been investigated to evaluate the potential exposure-associated risks. We observe silver accumulation in lungs 24 h after inhalation without signs of inflammation or tissue damages. AgNAs show a good excretion profile, and an almost negligible accumulation in the main organs. Interestingly, AgNAs demonstrate a lower persistence as well as a different biodistribution with respect to AuNAs. These differences can be ascribed to the chemical nature of the metals. Indeed, the inertness of gold avoids its dissolution and redistribution as ions while silver can be dissolved by oxidation to Ag^+^. Nevertheless, additional investigations on chronic exposure are needed as well as further pulmonary end points. Our findings highlight that the design of nanomaterials is pivotal to modulate the nano/biointeractions. Overall, our data suggest that rationally designed hybrid silver nanomaterials might be exploited to develop future intranasal treatments for communicable and noncommunicable diseases of the bottom respiratory tract, ultimately favoring patient compliance.

## Supporting Information Available

Materials and methods, and additional TEM and histological analysis (PDF)

## References

[ref1] SCCS (Scientific Committee on Consumer Safety) Scientific advice on the safety of nanomaterials in cosmetics, preliminary version of 6 October 2020, final version of 8 January 2021, SCCS/1618/20, Corrigendum of 8 March 2021.

[ref2] Epstein M.; Emri I.; Hartemann P.; Hoet P.; Leitgeb N.; Martínez L.; Proykova A.; Rizzo L.; Rodriguez-Farré E. Opinion on the Guidance on the Determination of Potential Health Effects of Nanomaterials Used in Medical Devices. Final Opin. 2015, 1–77.

[ref3] Ferdous Z.; Al-Salam S.; Yuvaraju P.; Ali B. H.; Nemmar A. Remote Effects and Biodistribution of Pulmonary Instilled Silver Nanoparticles in Mice. NanoImpact 2021, 22, 100310 10.1016/j.impact.2021.100310. 35559967

[ref4] Tang S.; Zheng J. Antibacterial Activity of Silver Nanoparticles: Structural Effects. Adv. Healthc. Mater. 2018, 7 (13), 1701503 10.1002/adhm.201701503. 29808627

[ref5] Sanderson-Wells T. H. A Case of Puerperal Septicæmia Successfully Treated with Intravenous Injections of Collosol Argentum. Lancet 1918, 191 (4929), 258–259. 10.1016/S0140-6736(01)23965-8.

[ref6] Eleraky N. E.; Allam A.; Hassan S. B.; Omar M. M. Nanomedicine Fight against Antibacterial Resistance: An Overview of the Recent Pharmaceutical Innovations. Pharmaceutics 2020, 12 (2), 142 10.3390/pharmaceutics12020142. PMC7076477 32046289

[ref7] Pilaquinga F.; Morey J.; Seqqat R.; De M.; Piña N. Silver Nanoparticles as a Potential Treatment against SARS-CoV-2: A Review. Wiley Interdiscip. Rev.: Nanomed. Nanobiotechnol. 2021, 13 (5), 1–19. 10.1002/wnan.1707. PMC7995207 33638618

[ref8] Bourquin J.; Milosevic A.; Hauser D.; Lehner R.; Blank F.; Petri-Fink A.; Rothen-Rutishauser B. Biodistribution, Clearance, and Long-Term Fate of Clinically Relevant Nanomaterials. Adv. Mater. 2018, 30 (19), 1704307 10.1002/adma.201704307. 29389049

[ref9] Li S.-C. Factors Affecting Therapeutic Compliance: A Review from the Patient’s Perspective. Ther. Clin. Risk Manag. 2008, 4 (1), 269–286. 10.2147/TCRM.S1458. 18728716 PMC2503662

[ref10] Wen R.; Hu L.; Qu G.; Zhou Q.; Jiang G. Exposure, Tissue Biodistribution, and Biotransformation of Nanosilver. NanoImpact 2016, 2, 18–28. 10.1016/j.impact.2016.06.001.

[ref11] Ferdous Z.; Nemmar A. Health Impact of Silver Nanoparticles: A Review of the Biodistribution and Toxicity Following Various Routes of Exposure. Int. J. Mol. Sci. 2020, 21 (7), 2375 10.3390/ijms21072375. PMC7177798 32235542

[ref12] Weldon B. A.; M. Faustman E.; Oberdörster G.; Workman T.; Griffith W. C.; Kneuer C.; Yu I. J. Occupational Exposure Limit for Silver Nanoparticles: Considerations on the Derivation of a General Health-Based Value. Nanotoxicology 2016, 10 (7), 945–956. 10.3109/17435390.2016.1148793. 26982810 PMC12091348

[ref13] Sung J. H.; Ji J. H.; Park J. D.; Yoon J. U.; Kim D. S.; Jeon K. S.; Song Y.; Jeong J.; Han S.; Han H.; Chung H.; Chang K.; Lee J. H.; Cho M. H.; Kelman B. J.; Yu I. J. Subchronic Inhalation Toxicity of Silver Nanoparticles. Toxicol. Sci. 2009, 108 (2), 452–461. 10.1093/toxsci/kfn246. 19033393

[ref14] Sung J. H.; Ji J. H.; Yoon J. U.; Kim D. S.; Song M. Y.; Jeong J.; Han B. S.; Han J. H.; Chung Y. H.; Kim J.; Kim T. S.; Chang H. K.; Lee E. J.; Lee J. H.; Yu I. J. Lung Function Changes in Sprague-Dawley Rats After Prolonged Inhalation Exposure to Silver Nanoparticles. Inhal. Toxicol. 2008, 20, 567–574. 10.1080/08958370701874671. 18444009

[ref15] Hadrup N.; Sharma A. K.; Loeschner K.; Jacobsen N. R. Pulmonary Toxicity of Silver Vapours, Nanoparticles and Fine Dusts: A Review. Regul. Toxicol. Pharmacol. 2020, 115 (March), 104690 10.1016/j.yrtph.2020.104690. 32474071

[ref16] Yu S. J.; Yin Y. G.; Liu J. F. Silver Nanoparticles in the Environment. Environ. Sci. Process. Impacts 2013, 15 (1), 78–92. 10.1039/C2EM30595J. 24592429

[ref17] Reidy B.; Haase A.; Luch A.; Dawson K. A.; Lynch I. Mechanisms of Silver Nanoparticle Release, Transformation and Toxicity: A Critical Review of Current Knowledge and Recommendations for Future Studies and Applications. Materials (Basel). 2013, 6 (6), 2295–2350. 10.3390/ma6062295. 28809275 PMC5458943

[ref18] Interaction of Nanomaterials with the Immune System; Bonner J. C., Brown J. M., Eds.; Springer: Cham, 2020.

[ref19] Wen R.; Yang X.; Hu L.; Sun C.; Zhou Q.; Jiang G. Brain-Targeted Distribution and High Retention of Silver by Chronic Intranasal Instillation of Silver Nanoparticles and Ions in Sprague - Dawley Rats. J. Appl. Toxicol. 2016, 36, 445–453. 10.1002/jat.3260. 26584724

[ref20] Hu Q.; Bai X.; Hu G.; Zuo Y. Y. Unveiling the Molecular Structure of Pulmonary Surfactant Corona on Nanoparticles. ACS Nano 2017, 11 (7), 6832–6842. 10.1021/acsnano.7b01873. 28541666

[ref21] Lee J. H.; Kim Y. S.; Song K. S.; Ryu H. R.; Sung J. H.; Park J. D.; Park H. M.; Song N. W.; Shin B. S.; Marshak D.; Ahn K.; Lee J. E.; Yu I. J. Biopersistence of Silver Nanoparticles in Tissues from Sprague-Dawley Rats. Part. Fibre Toxicol. 2013, 10 (1), 1–14. 10.1186/1743-8977-10-36. 24059869 PMC3734196

[ref22] van der Zande M.; Vandebriel R. J.; Van Doren E.; Kramer E.; Herrera Rivera Z.; Serrano-Rojero C. S.; Gremmer E. R.; Mast J.; Peters R. J. B.; Hollman P. C. H.; Hendriksen P. J. M.; Marvin H. J. P.; Peijnenburg A. A. C. M.; Bouwmeester H. Distribution, Elimination, and Toxicity of Silver Nanoparticles and Silver Ions in Rats after 28-Day Oral Exposure. ACS Nano 2012, 6 (8), 7427–7442. 10.1021/nn302649p. 22857815

[ref23] SCENIHR (Scientific Committee on Emerging and Newly Identified Health Risks). Nanosilver: safety, health and environmental effects and role in antimicrobial resistance; 2014, https://ec.europa.eu/health/publications/nanosilver-safety-health-and-environmental-effects-and-role-antimicrobial-resistance_en.

[ref24] Herzog F.; Clift M. J. D.; Piccapietra F.; Behra R.; Schmid O.; Petri-Fink A.; Rothen-Rutishauser B. Exposure of Silver-Nanoparticles and Silver-Ions to Lung Cells *in Vitro* at the Air-Liquid Interface. Part. Fibre Toxicol. 2013, 10 (1), 1–14. 10.1186/1743-8977-10-11. 23557437 PMC3639923

[ref25] Pernakov M.; Ermini M. L.; Sulaieva O.; Cassano D.; Santucci M.; Husak Y.; Korniienko V.; Giannone G.; Yusupova A.; Liubchak I.; Hristova M. T.; Savchenko A.; Holubnycha V.; Voliani V.; Pogorielov M. Complementary Effect of Non-Persistent Silver Nano-Architectures and Chlorhexidine on Infected Wound Healing. Biomedicines 2021, 9 (9), 1215 10.3390/biomedicines9091215. 34572402 PMC8469683

[ref26] Sarogni P.; Mapanao A. K.; Gonnelli A.; Ermini M. L.; Marchetti S.; Kusmic C.; Paiar F.; Voliani V. Chorioallantoic Membrane Tumor Models Highlight the Effects of Cisplatin Compounds in Oral Carcinoma Treatment. iScience 2022, 25, 103980 10.1016/j.isci.2022.103980. 35310338 PMC8924639

[ref27] Cassano D.; Mapanao A.-K.; Summa M.; Vlamidis Y.; Giannone G.; Santi M.; Guzzolino E.; Pitto L.; Poliseno L.; Bertorelli R.; Voliani V. Biosafety and Biokinetics of Noble Metals: The Impact of Their Chemical Nature. ACS Appl. Bio Mater. 2019, 2 (10), 4464–4470. 10.1021/acsabm.9b00630. 35021406

[ref28] Mapanao A. K.; Giannone G.; Summa M.; Ermini M. L.; Zamborlin A.; Santi M.; Cassano D.; Bertorelli R.; Voliani V. Biokinetics and Clearance of Inhaled Gold Ultrasmall-in-Nano Architectures. Nanoscale Adv. 2020, 2 (9), 3815–3820. 10.1039/D0NA00521E. PMC9417912 36132776

[ref29] Cassano D.; Summa M.; Pocoví-Martínez S.; Mapanao A.-K.; Catelani T.; Bertorelli R.; Voliani V. Biodegradable Ultrasmall-in-Nano Gold Architectures: Mid-Period *In Vivo* Distribution and Excretion Assessment. Part. Part. Syst. Charact. 2019, 36 (2), 1800464 10.1002/ppsc.201800464.

[ref30] Pocovı-Martınez S.; Cassano D.; Voliani V. Naked Nanoparticles in Silica Nanocapsules: A Versatile Family of Nanorattle Catalysts. ACS Appl. Nano Mater. 2018, 1 (4), 1836–1840. 10.1021/acsanm.8b00247.

[ref31] Armanetti P.; Pocoví-Martínez S.; Flori A.; Avigo C.; Cassano D.; Menichetti L.; Voliani V. Dual Photoacoustic/Ultrasound Multi-Parametric Imaging from Passion Fruit-like Nano-Architectures. Nanomedicine Nanotechnology, Biol. Med. 2018, 14 (6), 1787–1795. 10.1016/j.nano.2018.05.007. 29778890

[ref32] Mapanao A. K.; Santi M.; Faraci P.; Cappello V.; Cassano D.; Voliani V. Endogenously Triggerable Ultrasmall-in-Nano Architectures: Targeting Assessment on 3D Pancreatic Carcinoma Spheroids. ACS Omega 2018, 3 (9), 11796–11801. 10.1021/acsomega.8b01719. 30320273 PMC6173554

[ref33] Santi M.; Mapanao A. K.; Cassano D.; Vlamidis Y.; Cappello V.; Voliani V. Endogenously-Activated Ultrasmall-in-Nano Therapeutics: Assessment on 3D Head and Neck Squamous Cell Carcinomas. Cancers (Basel). 2020, 12 (5), 1063 10.3390/cancers12051063. PMC7281743 32344838

[ref34] Avigo C.; Cassano D.; Kusmic C.; Voliani V.; Menichetti L. Enhanced Photoacoustic Signal of Passion Fruit-Like Nanoarchitectures in a Biological Environment. J. Phys. Chem. C 2017, 121 (12), 6955–6961. 10.1021/acs.jpcc.6b11799.

[ref35] Mapanao A. K.; Santi M.; Voliani V. Combined Chemo-Photothermal Treatment of Three-Dimensional Head and Neck Squamous Cell Carcinomas by Gold Nano-Architectures. J. Colloid Interface Sci. 2021, 582, 1003–1011. 10.1016/j.jcis.2020.08.059. 32927167

[ref36] Wu L.; Rodríguez-Rodríguez C.; Cun D.; Yang M.; Saatchi K.; Häfeli U. O. Quantitative Comparison of Three Widely-Used Pulmonary Administration Methods *in Vivo* with Radiolabeled Inhalable Nanoparticles. Eur. J. Pharm. Biopharm. 2020, 152, 108–115. 10.1016/j.ejpb.2020.05.004. 32437751

[ref37] Wolff R. K. Toxicology Studies for Inhaled and Nasal Delivery. Mol. Pharmaceutics 2015, 12 (8), 2688–2696. 10.1021/acs.molpharmaceut.5b00146. 25915006

[ref38] Abdellatif A. A. H.; Khan R. A.; Alhowail A. H.; Alqasoumi A.; Sajid S. M.; Mohammed A. M.; Alsharidah M.; Al Rugaie O.; Mousa A. M. Octreotide-Conjugated Silver Nanoparticles for Active Targeting of Somatostatin Receptors and Their Application in a Nebulized Rat Model. Nanotechnol. Rev. 2022, 11 (1), 266–283. 10.1515/ntrev-2022-0021.

[ref39] Davidson R. A.; Anderson D. S.; Van Winkle L. S.; Pinkerton K. E.; Guo T. Evolution of Silver Nanoparticles in the Rat Lung Investigated by X-ray Absorption Spectroscopy. J. Phys. Chem. A 2015, 119, 281–289. 10.1021/jp510103m. 25517690 PMC4298353

[ref40] Rosário F.; Duarte I. F.; Pinto R. J. B.; Santos C.; Hoet P. H. M.; Oliveira H. Biodistribution and Pulmonary Metabolic Effects of Silver Nanoparticles in Mice Following Acute Intratracheal Instillations. Environ. Sci. Pollut. Res. 2021, 28 (2), 2301–2314. 10.1007/s11356-020-10563-z. 32885333

[ref41] Andriamasinoro S. N.; Dieme D.; Marie-Desvergne C.; Serventi A. M.; Debia M.; Haddad S.; Bouchard M. Kinetic Time Courses of Inhaled Silver Nanoparticles in Rats. Arch. Toxicol. 2022, 96, 487–498. 10.1007/s00204-021-03191-0. 34787690

[ref42] Ostrowski L. E.; Bennett W. D. Cilia and Mucociliary Clearance. Encycl. Respir. Med. Four-Volume Set 2006, 466–470. 10.1016/B0-12-370879-6/00079-X.

[ref43] Keller L. A.; Merkel O.; Popp A. Intranasal Drug Delivery: Opportunities and Toxicologic Challenges during Drug Development. Drug Delivery Transl. Res. 2022, 12 (4), 735–757. 10.1007/s13346-020-00891-5. PMC7829061 33491126

[ref44] Matsuo K.; Palmer J. B. Mastication, Swallowing and Breathing. Jpn. Dent. Sci. Rev. 2009, 45 (1), 31–40. 10.1016/j.jdsr.2009.03.004. 20161022 PMC2749282

[ref45] Chen X.; Schluesener H. J. Nanosilver: A Nanoproduct in Medical Application. Toxicol. Lett. 2008, 176 (1), 1–12. 10.1016/j.toxlet.2007.10.004. 18022772

[ref46] Kim Y. S.; Song M. Y.; Park J. D.; Song K. S.; Ryu H. R.; Chung Y. H.; Chang H. K.; Lee J. H.; Oh K. H.; Kelman B. J.; Hwang I. K.; Yu I. J. Subchronic Oral Toxicity of Silver Nanoparticles. Part. Fibre Toxicol. 2010, 7 (7), 20 10.1186/1743-8977-7-20. 20691052 PMC2928176

[ref47] Loeschner K.; Hadrup N.; Qvortrup K.; Larsen A.; Gao X.; Vogel U.; Mortensen A.; Lam H. R.; Larsen E. H. Distribution of Silver in Rats Following 28 Days of Repeated Oral Exposure to Silver Nanoparticles or Silver Acetate. Part. Fibre Toxicol. 2011, 8, 18 10.1186/1743-8977-8-18. 21631937 PMC3123173

[ref48] Aird W. C. Phenotypic Heterogeneity of the Endothelium: I. Structure, Function, and Mechanisms. Circ. Res. 2007, 100 (2), 158–173. 10.1161/01.RES.0000255691.76142.4a. 17272818

[ref49] Wisse E.; Jacobs F.; Topal B.; Frederik P.; De Geest B. The Size of Endothelial Fenestrae in Human Liver Sinusoids: Implications for Hepatocyte-Directed Gene Transfer. Gene Ther. 2008, 15 (17), 1193–1199. 10.1038/gt.2008.60. 18401434

[ref50] Holland N.; Becak D.; Shannahan J. H.; Brown J.; Carratt S.; Winkle L.; Pinkerton K.; Wang C.; Munusamy P.; Baer D. R.; Sumner S.; Fennell T.; Lust R.; Wingard C. Cardiac Ischemia Reperfusion Injury Following Instillation of 20 Nm Citrate-Capped Nanosilver. J. Nanomed. Nanotechnol. 2015, 6 (Suppl 6), 1–28. 10.4172/2157-7439.S6-006 PMC4780684 26966636

[ref51] Ferdous Z.; Al-Salam S.; Greish Y. E.; Ali B. H.; Nemmar A. Pulmonary Exposure to Silver Nanoparticles Impairs Cardiovascular Homeostasis: Effects of Coating, Dose and Time. Toxicol. Appl. Pharmacol. 2019, 367, 36–50. 10.1016/j.taap.2019.01.006. 30639276

[ref52] Rahman M. F.; Wang J.; Patterson T. A.; Saini U. T.; Robinson B. L.; Newport G. D.; Murdock R. C.; Schlager J. J.; Hussain S. M.; Ali S. F. Expression of Genes Related to Oxidative Stress in the Mouse Brain after Exposure to Silver-25 Nanoparticles. Toxicol. Lett. 2009, 187 (1), 15–21. 10.1016/j.toxlet.2009.01.020. 19429238

[ref53] Liu Y.; Guan W.; Ren G.; Yang Z. The Possible Mechanism of Silver Nanoparticle Impact on Hippocampal Synaptic Plasticity and Spatial Cognition in Rats. Toxicol. Lett. 2012, 209 (3), 227–231. 10.1016/j.toxlet.2012.01.001. 22245254

[ref54] Jonas L.; Bloch C.; Zimmermann R.; Stadie V.; Gross G. E.; Schäd S. G. Detection of Silver Sulfide Deposits in the Skin of Patients with Argyria after Long-Term Use of Silver-Containing Drugs. Ultrastruct. Pathol. 2007, 31 (6), 379–384. 10.1080/01913120701696221. 18098055

[ref55] Wegener H.; Paulsen H.; Seeger K. The Cysteine-Rich Region of Type VII Collagen Is a Cystine Knot with a New Topology. J. Biol. Chem. 2014, 289 (8), 4861–4869. 10.1074/jbc.M113.531327. 24385431 PMC3931048

[ref56] Liu J.; Wang Z.; Liu F. D.; Kane A. B.; Hurt R. H. Chemical Transformations of Nanosilver in Biological Environments. ACS Nano 2012, 6 (11), 9887–9899. 10.1021/nn303449n. 23046098 PMC3508364

[ref57] Kim Y. S.; Kim J. S.; Cho H. S.; Rha D. S.; Kim J. M.; Park J. D.; Choi B. S.; Lim R.; Chang H. K.; Chung Y. H.; Kwon I. H.; Jeong J.; Han B. S.; Yu I. J. Twenty-Eight-Day Oral Toxicity, Genotoxicity, and Gender-Related Tissue Distribution of Silver Nanoparticles in Sprague-Dawley Rats. Inhal. Toxicol. 2008, 20 (6), 575–583. 10.1080/08958370701874663. 18444010

[ref58] Poley M.; Mora-Raimundo P.; Shammai Y.; Kaduri M.; Koren L.; Adir O.; Shklover J.; Shainsky-Roitman J.; Ramishetti S.; Man F.; de Rosales R. T. M.; Zinger A.; Peer D.; Ben-Aharon I.; Schroeder A. Nanoparticles Accumulate in the Female Reproductive System during Ovulation Affecting Cancer Treatment and Fertility. ACS Nano 2022, 16, 5246–5257. 10.1021/acsnano.1c07237. PMC7613117 35293714

[ref59] Moradi-Sardareh H.; Basir H. R. G.; Hassan Z. M.; Davoudi M.; Amidi F.; Paknejad M. Toxicity of Silver Nanoparticles on Different Tissues of Balb/C Mice. Life Sci. 2018, 211, 81–90. 10.1016/j.lfs.2018.09.001. 30189219

[ref60] Lansdown A. B. G. A Pharmacological and Toxicological Profile of Silver as an Antimicrobial Agent in Medical Devices. Adv. Pharmacol. Sci. 2010, 2010, 910686. 21188244 10.1155/2010/910686 PMC3003978

[ref61] Ballatori N. Glutathione Mercaptides as Transport Forms of Metals. Adv. Pharmacol. 1994, 27 (C), 271–298. 10.1016/S1054-3589(08)61036-4. 8068556

[ref62] Braakhuis H. M.; Gosens I.; Krystek P.; Boere J. A. F.; Cassee F. R.; Fokkens P. H. B.; Post J. A.; van Loveren H.; Park M. V. D. Z. Particle Size Dependent Deposition and Pulmonary Inflammation after Short-Term Inhalation of Silver Nanoparticles. Part. Fibre Toxicol. 2014, 11, 49 10.1186/s12989-014-0049-1. 25227272 PMC4410796

[ref63] Smulders S.; Larue C.; Sarret G.; Castillo-Michel H.; Vanoirbeek J.; Hoet P. H. M. Lung Distribution, Quantification, Co-Localization and Speciation of Silver Nanoparticles after Lung Exposure in Mice. Toxicol. Lett. 2015, 238 (1), 1–6. 10.1016/j.toxlet.2015.07.001. 26162856

[ref64] Oberdörster G.; Sharp Z.; Atudorei V.; Elder A.; Gelein R.; Kreyling W.; Cox C. Translocation of Inhaled Ultrafine Particles to the Brain. Inhal. Toxicol. 2004, 16 (6–7), 437–445. 10.1080/08958370490439597. 15204759

