# Supplementary material for: The Fate of Intranasally Instilled Silver Nanoarchitectures
Source: Nano Lett. 2022 Jun 30;22(13):5269–76. doi: 10.1021/acs.nanolett.2c01180 (PMC9284613; doi:10.1021/acs.nanolett.2c01180)
Supplement: Supplementary file 1 — nl2c01180_si_001.pdf [file nl2c01180_si_001.pdf]

Supporting information

The fate of intranasally instilled silver nano-architectures

Agata Zamborlin,[1,2] Maria Laura Ermini,[1] Maria Summa,[3] Giulia Giannone,[1,2] Valentina Frusca,[1] Ana Katrina Mapanao,[1] Doriana Debellis,[4] Rosalia Bertorelli,[3] and Valerio Voliani[1,*]

[1] Center for Nanotechnology Innovation@ NEST, Istituto Italiano di Tecnologia, Piazza San Silvestro, 12 – 56127, Pisa, Italy
[2] NEST-Scuola Normale Superiore, Piazza San Silvestro, 12 – 56127, Pisa, Italy
[3] Translational Pharmacology, Istituto Italiano di Tecnologia, Via Morego, 30 – 16163, Genoa, Italy
[4] Electron Microscopy Facility, Istituto Italiano di Tecnologia, Via Morego, 30 – 16163, Genoa, Italy

Corresponding Author: valerio.voliani@iit.it

Materials & Methods

Materials. All chemicals were purchased from Sigma-Aldrich, unless differently specified, and used as received. Animals were purchased by Charles River Laboratories (Calco, Italy).

Synthesis of Silver nano-architectures (AgNAs). Silver ultrasmall nanoparticles (USNPs) were prepared by adding in 4°C 20 mL of Milli-Q® water, in order, 200 µL of L-glutathione reduced (GSH) (cold aqueous solution, 100 mM) and 200 µL of $AgNO_3$ cold aqueous solution (25 mM). During vigorous stirring, 200 µL of $NaBH_4$ cold aqueous solution (211 mM) was added. After 2 minutes, the solution became light yellow, and it was let under vigorous stirring for 30 minutes in ice-bath. Then, 10 µL of 30% poly(sodium 4-styrenesulfonate) (PSS) was added, and the mixture was left under stirring for 15 minutes at room temperature. Under mild stirring, 75 µL of poly(L-lysine) (PL, 40 mg/mL) was slowly added, and the mixture was further left under agitation for 15 minutes. Aggregates were collected by 3-minute centrifugation at 17092 rcf and resuspended in 6.5 mL of Milli-Q® water. The solution was added to 70 mL of absolute ethanol previously added of 60 µL of tetraethyl orthosilicate (TEOS, 98%). After 10 minutes of gently shaking, 141 µL of dimethylamine (DMA, 40% stock) was added, and the mixture was left under gentle shaking for 3 hours at room temperature. AgNAs were collected by 30-minute centrifugation at 3220 rcf and resuspended in 2 mL of ethanol. NAs were washed once with ethanol and once with water to remove unreacted precursors. Bigger AgNAs were eliminated

through a short spin (14 seconds at 14462 rcf) and the recovered supernatant was washed once more in ethanol and stored in 1 mL of ethanol at -20°C.

Electron Microscopy. Transmission electron microscopy (TEM) images were taken using ZEISS Libra 120, operated at 120 kV accelerating voltage. An AgNAs suspension was dropped on 300-mesh carbon-coated copper grids and dried before observation. TEM images were analysed using ImageJ. Particle diameters and silica shell were measured on at least 100 nanoparticles.
Animals were perfused with 2% glutaraldehyde and 4% paraformaldehyde in 0.1 M sodium-cacodylate buffer. Only a portion of the lung was examined, specifically the inferior lobe since the other portions were utilized for the other analysis. The studies were conducted in 3 mice 24 hours after AgNAs administration. Tissues were collected and fixed with 1.5% glutaraldehyde in 0.1 M cacodylate buffer and, after several washings in the same buffer, were post-fixed in 1% osmium tetroxide in 0.1 M cacodylate buffer for 2 hours. Then, tissues were stained overnight at 4°C in an aqueous 1% uranyl acetate solution. After several washings in Milli-Q® water, the samples were dehydrated in a graded ethanol series and embedded in SPURR resin. Sections of about 70 nm were cut with a diamond knife on a Leica EM UC6 ultramicrotome. TEM images were collected with a Jeol JEM 1011 (Jeol, Japan) electron microscope and recorded with a 2 Mp charge-coupled device camera (Gatan Orius SC100).

Inductively Coupled Plasma–Mass Spectrometry (ICP–MS) Analysis. The quantification of the mass of silver encapsulated in AgNAs was performed using an ICP–MS Agilent 7700 (Agilent Technologies, Santa Clara, CA, USA). Samples were pre-digested in nitric acid (95% Suprapur®) and stirred at 200°C under microwave irradiation with CEM Discover SP-D digestion microwave (CEM, Matthews, NC, USA). The resulting solution was diluted in 3 mL of 3% nitric acid solution, and the Ag content was assessed by analysis against a standard calibration curve with 10 ppm Hg in 3% nitric acid solution as internal standard.
Samples from mice organs and excretions were first dried overnight at 80°C until a constant weight was reached. Samples were then transferred to 10 mL pressure vessels and digested in nitric acid at 150°C for 30 minutes. Lastly, samples were dried and diluted with 3% nitric acid solution to 2.5 mL. The silver amount was determined after analysis on ICP-MS Agilent 7700, using standard calibration curve.

***In vivo* experiments.** Male CD1-*Foxn1*^{nu} mice, 8 weeks old (Charles River, Calco, Italy), were used for *in vivo* tests. The animals were group-housed in ventilated cages and had free access to food and water. They were maintained under a 12 hour-light/dark cycle (lights on at 8:00 am) at a controlled temperature of 21±1°C and relative humidity of 55±10% for one week. After acclimation, the animals were housed singularly in metabolic cages, in order to collect urine and faeces, for 24h, 48h and 72h (*n*=3 per group). All experiments were carried out in accordance

with the guidelines established by the European Communities Council Directive (Directive 2010/63/EU of 22 September 2010) and approved by the National Council on Animal Care of the Italian Ministry of Health (authorization: 686/2019-PR). All efforts were made to minimize animal suffering and to use the lowest possible number of animals required to produce statistically relevant results, according to the “3Rs concept”. Only for the experimental period, the mice were housed in metabolic cages and monitored daily for body weight, food and water intake, and urine and faeces output. NAs were resuspended in sterile saline to reach the final concentration of 3 mg NAs/kg mouse. CD1-*Foxn1*^{nu} male mice were anesthetized with intraperitoneal ketamine and xylazine (100 and 10 mg/kg, respectively). Mice were intranasal administered with 20 µL of NAs dissolved in saline into both nares by using a pipette. At the end of experiments, mice were sacrificed. Tissues were collected, snap-frozen in liquid nitrogen and stored at 80°C until required for subsequent measurements, such as ICP-MS, TEM, and histological analysis.

Histology. Tissues for histological examination were fixed in 10% formalin solution, embedded in paraffin and serial sections were collected. Sections were stained with hematoxylin and eosin (H&E) and analysed with a Leica DM5500 optical microscope (*n*=3 each group).

Figure S1

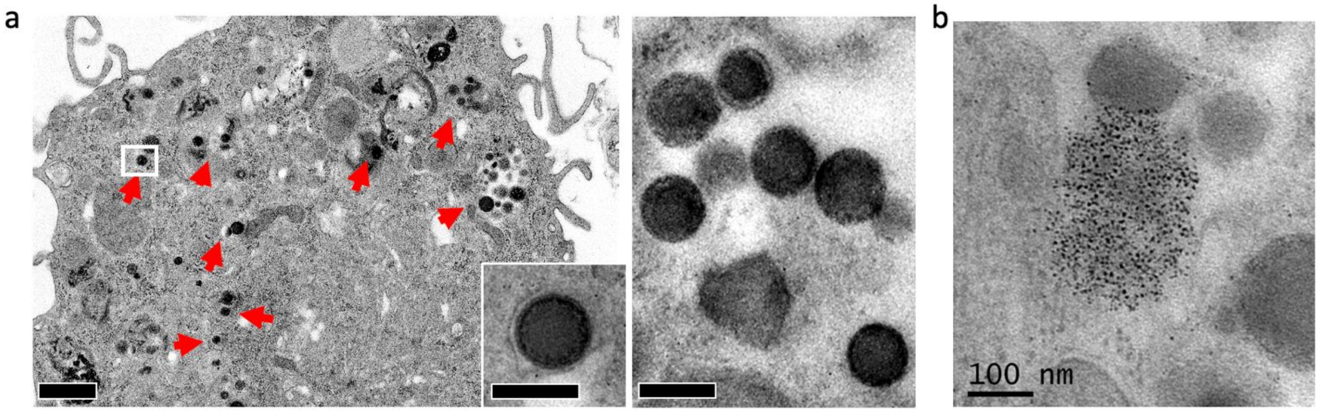

Figure S1. a) TEM micrographs showing the presence of AgNAs in lungs (red arrows) 24 hours after administration, and details of AgNAs. Scale bar: 1 µm, and (for the zoom on the single AgNAs) 200 nm. b) TEM micrograph showing Ag USNPs in the cytosol.

Figure S2

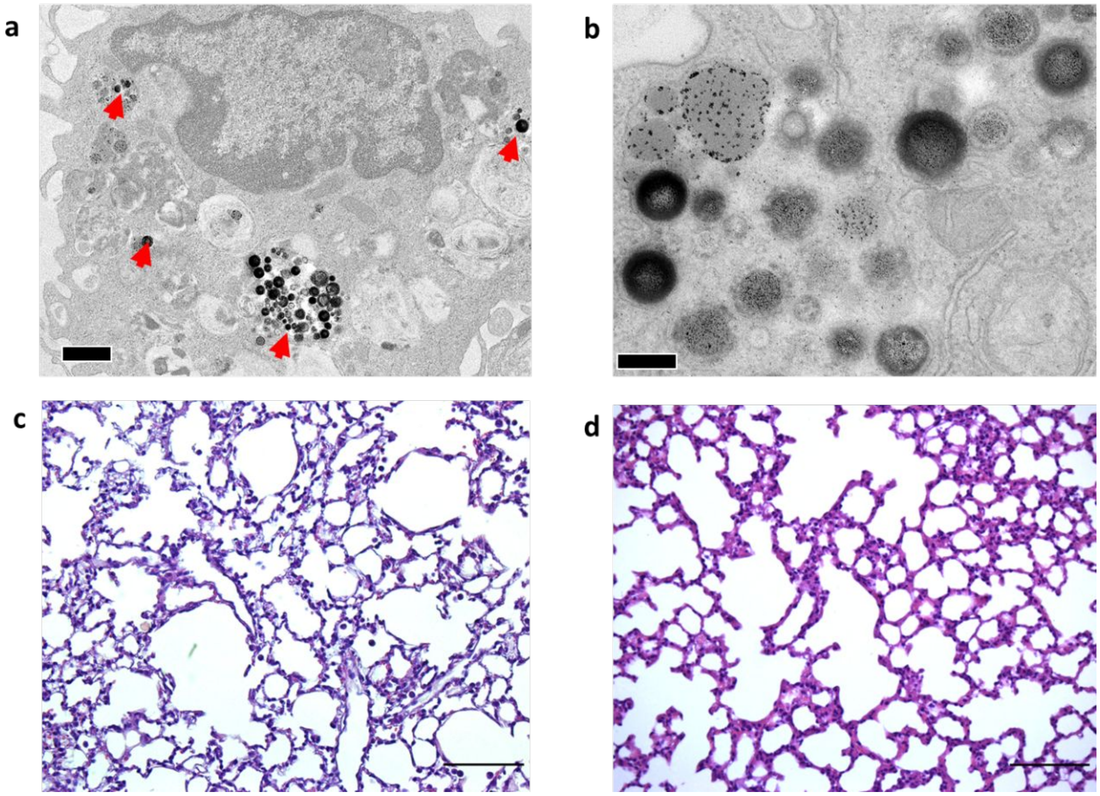

Figure S2. TEM micrograph showing a) the presence of intact AuNAs in lungs (red arrows); b) details of AuNAs with a partially or completely degraded silica shell. Scale bar is a) 1 µm and b) 200 nm. Histological analysis of lung tissues of control (c), and IN AuNPs (d) treated mice. Scale bar is 100 µm.
